# Potentials of Mean Force for Protein Structure Prediction Vindicated, Formalized and Generalized

**DOI:** 10.1371/journal.pone.0013714

**Published:** 2010-11-10

**Authors:** Thomas Hamelryck, Mikael Borg, Martin Paluszewski, Jonas Paulsen, Jes Frellsen, Christian Andreetta, Wouter Boomsma, Sandro Bottaro, Jesper Ferkinghoff-Borg

**Affiliations:** 1 Bioinformatics Center, Department of Biology, University of Copenhagen, Copenhagen, Denmark; 2 Biomedical Engineering, Technical University of Denmark (DTU) Elektro, Technical University of Denmark, Lyngby, Denmark; 3 Department of Chemistry, University of Cambridge, Cambridge, United Kingdom; University of Oxford, United Kingdom

## Abstract

Understanding protein structure is of crucial importance in science, medicine and biotechnology. For about two decades, knowledge-based potentials based on pairwise distances – so-called “potentials of mean force” (PMFs) – have been center stage in the prediction and design of protein structure and the simulation of protein folding. However, the validity, scope and limitations of these potentials are still vigorously debated and disputed, and the optimal choice of the reference state – a necessary component of these potentials – is an unsolved problem. PMFs are loosely justified by analogy to the reversible work theorem in statistical physics, or by a statistical argument based on a likelihood function. Both justifications are insightful but leave many questions unanswered. Here, we show for the first time that PMFs can be seen as approximations to quantities that do have a rigorous probabilistic justification: they naturally arise when probability distributions over different features of proteins need to be combined. We call these quantities “reference ratio distributions” deriving from the application of the “reference ratio method.” This new view is not only of theoretical relevance but leads to many insights that are of direct practical use: the reference state is uniquely defined and does not require external physical insights; the approach can be generalized beyond pairwise distances to arbitrary features of protein structure; and it becomes clear for which purposes the use of these quantities is justified. We illustrate these insights with two applications, involving the radius of gyration and hydrogen bonding. In the latter case, we also show how the reference ratio method can be iteratively applied to sculpt an energy funnel. Our results considerably increase the understanding and scope of energy functions derived from known biomolecular structures.

## Introduction

Methods for protein structure prediction, simulation and design rely on an energy function that represents the protein's free energy landscape; a protein's native state typically corresponds to the state with minimum free energy [1]. So-called knowledge based potentials (KBP) are parametrized functions for free energy calculations that are commonly used for modeling protein structures [2], [3]. These potentials are obtained from databases of known protein structures and lie at the heart of some of the best protein structure prediction methods. The use of KBPs originates from the work of Tanaka and Scheraga [4] who were the first to extract effective interactions from the frequency of contacts in X-ray structures of native proteins. Miyazawa and Jernigan formalized the theory for contact interactions by means of the quasi-chemical approximation [5], [6].

Many different approaches for developing KBPs exist, but the most successful methods to date build upon a seminal paper by Sippl – published two decades ago – which introduced KBPs based on probability distributions of pairwise distances in proteins and reference states [7]. These KBPs were called “potentials of mean force”, and seen as approximations of free energy functions. Sippl's work was inspired by the statistical physics of liquids, where a “potential of mean force” has a very precise and undisputed definition and meaning [8], [9]. However, the validity of the application to biological macromolecules is vigorously disputed in the literature [2], [10]–[17]. Nonetheless, PMFs are widely used with considerable success; not only for protein structure prediction [3], [18], [19], but also for quality assessment and identification of errors [20]–[22], fold recognition and threading [23], [24], molecular dynamics [24], protein-ligand interactions [16], [25], protein design and engineering [26], [27], and the prediction of binding affinity [17], [28]. In this article, the abbreviation “PMF” will refer to the pairwise distance dependent KBPs following Sippl [7], and the generalization that we introduce in this article; we will write “potentials of mean force” in full when we refer to the real, physically valid potentials as used in liquid systems [9], [13], [29]. At the end of the article, we will propose a new name for these statistical quantities, to set them apart from true potentials of mean force with a firm physical basis.

Despite the progress in methodology and theory, and the dramatic increase in the number of experimentally determined protein structures, the accuracy of the energy functions still remains the main obstacle to accurate protein structure prediction [22], [30], [31]. Recently, several groups demonstrated that it is the quality of the coarse grained energy functions [18], rather than inadequate sampling, that impairs the successful prediction of the native state [30], [31]. The insights presented in this article point towards a new, theoretically well-founded way to construct and refine energy functions, and thus address a timely problem.

We start with an informal outline of the general ideas presented in this article, and then analyze two notable attempts in the literature to justify PMFs. We point out their shortcomings, and subsequently present a rigorous probabilistic explanation of the strengths and shortcomings of traditional pairwise distance PMFs. This explanation sheds a surprising new light on the nature of the reference state, and allows the generalization of PMFs beyond pairwise distances in a statistically valid way. Finally, we demonstrate our method in two applications involving protein compactness and hydrogen bonding. In the latter case, we also show that PMFs can be iteratively optimized, thereby effectively sculpting an energy funnel [24], [32]–[36].

## Results and Discussion

### Overview

**Figure 1:**
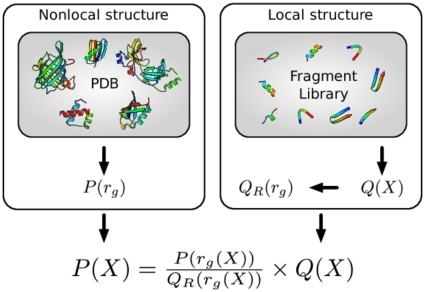
Illustration of the central idea presented in this article. In this example, the goal is to sample conformations with a given distribution 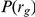 for the radius of gyration 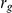, and a plausible local structure. 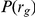 could, for example, be derived from known structures in the Protein Data Bank (PDB, left box). 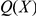 is a probability distribution over local structure 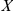, typically embodied in fragment library (right box). In order to combine 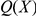 and 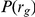 in a meaningful way (see text), the two distributions are multiplied and divided by 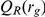 (formula at the bottom); 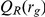 is the probability distribution over the radius of gyration for conformations sampled solely from the fragment library (that is, 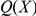). The probability distribution 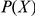 will generate conformations with plausible local structures (due to 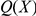), while their radii of gyration will be distributed according to 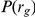, as desired. This simple idea lies at the theoretical heart of the PMF expressions used in protein structure prediction.

In order to emphasize the practical implications of the theoretical insights that we present here, we start with a very concrete example that illustrates the essential concepts (see Fig. 1). Currently, protein structure prediction methods often make use of fragment libraries: collections of short fragments derived from known protein structures in the Protein Data Bank (PDB). By assembling a suitable set of fragments, one obtains conformations that are protein-like on a local length scale. That is, these conformations typically lack non-local features that characterize real proteins, such as a well-packed hydrophobic core or an extensive hydrogen bond network. Such aspects of protein structure are not, or only partly, captured by fragment libraries.

Formally, a fragment library specifies a probability distribution 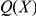, where 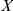 is for example a vector of dihedral angles. In order to obtain conformations that also possess the desired non-local features, 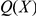 needs to be complemented with another probability distribution 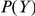, with 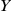 being for example a vector of pairwise distances, the radius of gyration, the hydrogen bonding network, or any combination of non-local features. Typically, 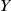 is a deterministic function of 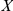; we use the notation 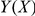 when necessary.

For the sake of argument, we will focus on the radius of gyration (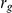) at this point; in this case 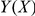 becomes 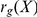. We assume that a suitable 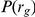 was derived from the set of known protein structures; without loss of generality, we leave out the dependency on the amino acid sequence for simplicity. The problem that we address in this article can be illustrated with the following question: how can we combine 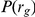 and 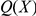 in a rigorous, meaningful way? In other words, we want to use the fragment library to sample conformations whose radii of gyration 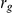 are distributed according to 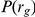. These conformations should display a realistic *local* structure as well, reflecting the use of the fragment library. Simply multiplying 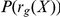 and 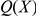 does not lead to the desired result, as 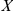 and 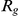 are not independent; the resulting conformations will not be distributed according to 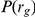.

The solution is given in Fig. 1; it involves the probability distribution 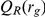, the probability distribution over the radius of gyration for conformations sampled solely from the fragment library. The subscript 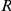 stands for *reference state* as will be explained below. The solution generates conformations whose radii of gyration are distributed according to 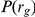. The influence of 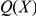 is apparent in the fact that for conformations with a given 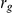, their local structure 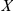 will be distributed according to 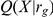. The latter distribution has a clear interpretation: it corresponds to sampling an infinite amount of conformations from a fragment library, and retaining only those with the desired 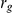. Note that even if we chose the uniform distribution for 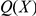, the resulting 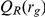 will *not* (necessarily) be uniform.

Intuitively, 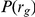 provides correct information about the radius of gyration, but no information about local structure; 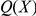 provides approximately correct information about the structure of proteins on a local length scale, but is incorrect on a global scale (leading to an incorrect probability distribution for the radius of gyration); finally, the formula shown in Fig. 1 merges these two complementary sources of information together. Another viewpoint is that 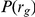 and 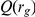 are used to correct the shortcomings of 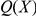. This construction is statistically rigorous, provided that 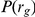 and 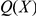 are proper probability distributions.

After this illustrative example, we now review the use of PMFs in protein structure prediction, and discuss how PMFs can be understood and generalized in the theoretical framework that we briefly outlined here.

### Pairwise PMFs for protein structure prediction

Many textbooks present PMFs as a simple consequence of the Boltzmann distribution, as applied to pairwise distances between amino acids. This distribution, applied to a specific pair of amino acids, is given by:
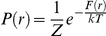
where 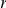 is the distance, 
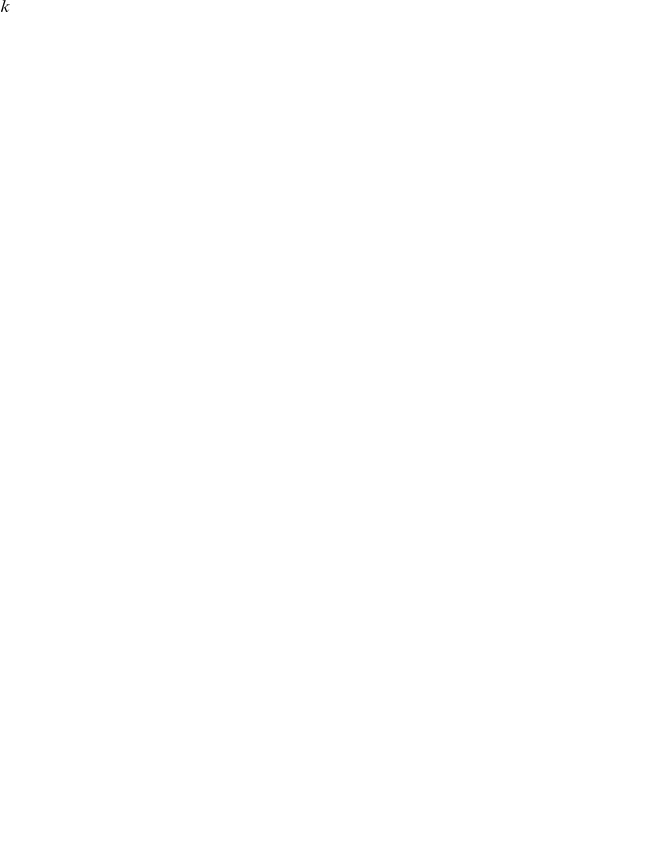
 is Boltzmann's constant, 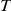 is the temperature and 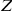 is the partition function, with 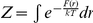. The quantity 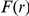 is the free energy assigned to the pairwise system. Simple rearrangement results in the *inverse Boltzmann formula*, which expresses the free energy 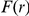 as a function of 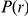:
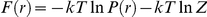
To construct a PMF, one then introduces a so-called *reference state* with a corresponding distribution 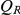 and partition function 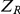, and calculates the following free energy difference:
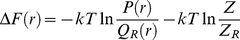
(1)


The reference state typically results from a hypothetical system in which the specific interactions between the amino acids are absent [7]. The second term involving 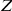 and 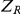 can be ignored, as it is a constant.

In practice, 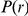 is estimated from the database of known protein structures, while 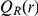 typically results from calculations or simulations. For example, 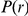 could be the conditional probability of finding the 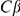 atoms of a valine and a serine at a given distance 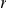 from each other, giving rise to the free energy difference 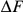. The total free energy difference of a protein, 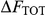, is then claimed to be the sum of all the pairwise free energies:
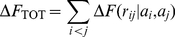
(2)

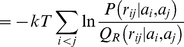
(3)where the sum runs over all amino acid pairs 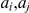 (with 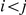) and 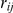 is their corresponding distance. It should be noted that in many studies 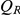 does not depend on the amino acid sequence [11].

Intuitively, it is clear that a low free energy difference indicates that the set of distances in a structure is more likely in proteins than in the reference state. However, the physical meaning of these PMFs have been widely disputed since their introduction [2], [12]–[15]. Indeed, why is it at all necessary to subtract a reference state energy? What is the optimal reference state? Can PMFs be generalized and justified beyond pairwise distances, and if so, how? Before we discuss and clarify these issues, we discuss two qualitative justifications that were previously reported in the literature: the first based on a physical analogy, and the second using a statistical argument.

### PMFs from the reversible work theorem

The first, qualitative justification of PMFs is due to Sippl, and based on an analogy with the statistical physics of liquids [37]. For liquids [8], [9], [13], [14], [37], the potential of mean force is related to the *pair correlation function* 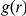, which is given by:
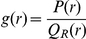
where 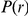 and 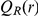 are the respective probabilities of finding two particles at a distance 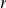 from each other in the liquid and in the reference state. For liquids, the reference state is clearly defined; it corresponds to the ideal gas, consisting of non-interacting particles. The two-particle potential of mean force 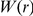 is related to 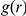 by:
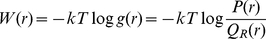
(4)According to the *reversible work theorem*, the two-particle potential of mean force 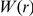 is the reversible work required to bring two particles in the liquid from infinite separation to a distance 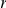 from each other [8], [9].

Sippl justified the use of PMFs – a few years after he introduced them for use in protein structure prediction [7] – by appealing to the analogy with the reversible work theorem for liquids [37]. For liquids, 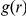 can be experimentally measured using small angle X-ray scattering; for proteins, 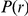 is obtained from the set of known protein structures, as explained in the previous section. The analogy described above might provide some physical insight, but, as Ben-Naim writes in a seminal publication [13]: “the quantities, referred to as ‘statistical potentials,’ ‘structure based potentials,’ or ‘pair potentials of mean force’, as derived from the protein data bank, are neither ‘potentials’ nor ‘potentials of mean force,’ in the ordinary sense as used in the literature on liquids and solutions.”

Another issue is that the analogy does not specify a suitable reference state for proteins. This is also reflected in the literature on statistical potentials; the construction of a suitable reference state continues to be an active research topic [3], [22], [38]–[41]. In the next section, we discuss a second, more recent justification that is based on probabilistic reasoning.

### PMFs from likelihoods

Baker and co-workers [18] justified PMFs from a Bayesian point of view and used these insights in the construction of the coarse grained ROSETTA energy function; Samudrala and Moult used similar reasoning for the RAPDF potential [42]. According to Bayesian probability calculus, the conditional probability 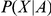 of a structure 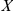, given the amino acid sequence 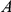, can be written as:
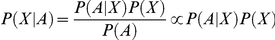


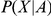
 is proportional to the product of the likelihood 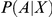 times the prior 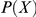. By assuming that the likelihood can be approximated as a product of pairwise probabilities, and applying Bayes' theorem, the likelihood can be written as:
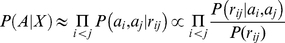
(5)where the product runs over all amino acid pairs 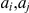 (with 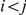), and 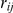 is the distance between amino acids 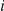 and 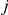. Obviously, the negative of the logarithm of expression (5) has the same functional form as the classic pairwise distance PMFs, with the denominator playing the role of the reference state in Eq. 1. The merit of this explanation is the qualitative demonstration that the functional form of a PMF can be obtained from probabilistic reasoning. Although this view is insightful – it rightfully drew the attention to the application of Bayesian methods to protein structure prediction – there is a more quantitative explanation, which does not rely on the incorrect assumption of pairwise decomposability [12]–[14], [43], and leads to a different, *quantitative* conclusion regarding the nature of the reference state. This explanation is given in the next section.

### A general statistical justification for PMFs

**Figure 2:**
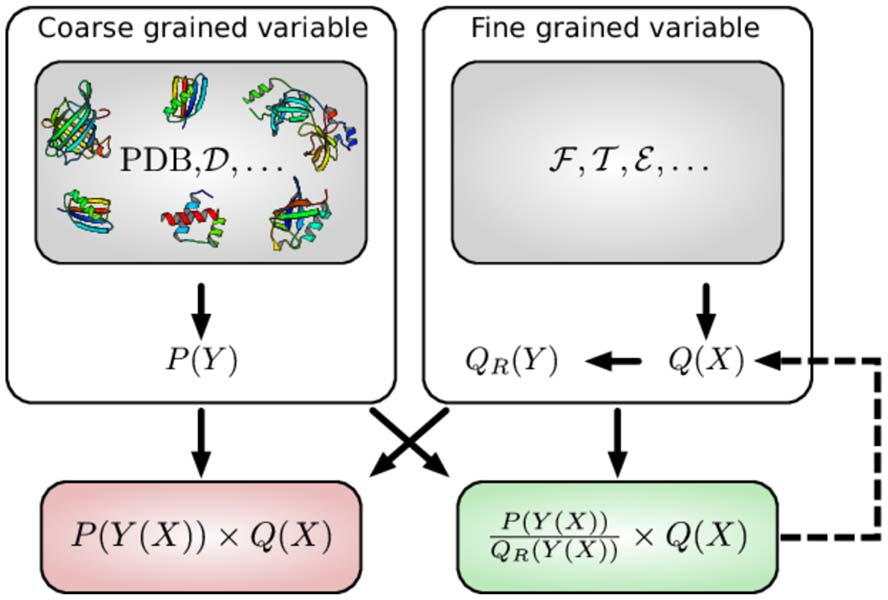
General statistical justification of PMFs. The goal is to combine a distribution 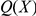 over a fine grained variable 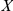 (top right), with a probability distribution 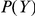 over a coarse grained variable 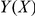 (top left). 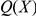 could be, for example, embodied in a fragment library (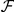), a probabilistic model of local structure (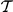) or an energy function (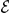); 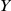 could be, for example, the radius of gyration, the hydrogen bond network, or the set of pairwise distances. 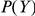 usually reflects the distribution of 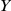 in known protein structures (PDB), but could also stem from experimental data (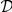). Sampling from 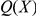 results in a distribution 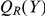 that differs from 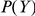. Multiplying 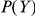 and 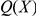 does not result in the desired distribution for 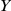 either (red box); the correct result requires dividing out the signal with respect to 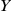 due to 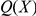 (green box). The *reference* distribution 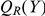 in the denominator corresponds to the contribution of the reference state in a PMF. If 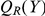 is only approximately known, the method can be applied iteratively (dashed arrow). In that case, one attempts to iteratively sculpt an energy funnel. The procedure is statistically rigorous provided 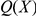 and 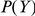 are proper probability distributions; this is usually not the case for conventional pairwise distance PMFs.

Expressions that resemble PMFs naturally result from the application of probability theory to solve a fundamental problem that arises in protein structure prediction: how to improve an imperfect probability distribution 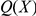 over a first variable 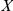 using a probability distribution 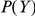 over a second variable 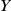 (see Fig. 2, Fig. 1 and Materials and Methods). We assume that 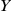 is a deterministic function of 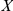; we write 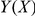 when necessary. In that case, 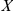 and 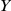 are called *fine* and *coarse grained variables*, respectively. When 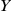 is a function of 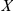, the probability distribution 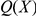 automatically implies a probability distribufotion 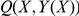. This distribution has some unusual properties: 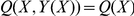; and if 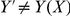, it follows that 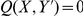.

Typically, 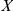 represents *local* features of protein structure (such as backbone dihedral angles), while 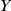 represents *nonlocal* features (such as hydrogen bonding, compactness or pairwise distances). However, the same reasoning also applies to other cases; for example, 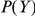 could represent information coming from experimental data, and 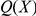 could be embodied in an empirical force field as used in molecular mechanics [2], [44] (see Fig. 2).

Typically, the distribution 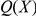 in itself is not sufficient for protein structure prediction: it does not consider important nonlocal features such as hydrogen bonding, compactness or favorable amino acid interactions. As a result, 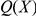 is incorrect with respect to 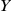, and needs to be supplemented with a probability distribution 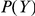 that provides additional information. By construction, 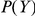 is assumed to be correct (or at least useful).

The above situation arises naturally in protein structure prediction. For example, 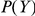 could be a probability distribution over the radius of gyration, hydrogen bond geometry or the set of pairwise distances, and 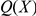 could be a fragment library [18] or a probabilistic model of local structure [45]. In Fig. 1, we used the example of a distribution over the radius of gyration for 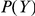 and a fragment library for 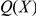. Obviously, sampling from a fragment library and retaining structures with the desired nonlocal structure (radius of gyration, hydrogen bonding, etc.) is in principle possible, but in practice extremely inefficient.

How can 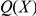 be combined with 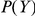 in a meaningful way? As mentioned previously, simply multiplying the two distributions – resulting in 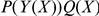 – does not lead to the desired result as the two variables are obviously not independent. The correct solution follows from simple statistical considerations (see Materials and Methods), and is given by the following expression:
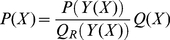
(6)We use the notation 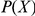, as this distribution implies the desired distribution 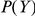 for 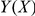. The distribution 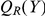 in the denominator is the probability distribution that is implied by 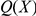 over the coarse grained variable 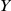. Conceptually, dividing by 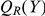 takes care of the signal in 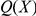 with respect to the coarse grained variable 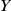. The ratio in this expression corresponds to the probabilistic formulation of a PMF, and 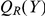 corresponds to the reference state (see Materials and Methods).

In practice, 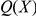 is typically not evaluated directly, but brought in through conformational Monte Carlo sampling (see Materials and Methods); often sampling is based on a fragment library [18], [46], although other methods are possible, including sampling from a probabilistic model [45], [47], [48] or a suitable energy function [2], [44]. The ratio 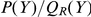, which corresponds to the probabilistic formulation of a PMF, also naturally arises in the Markov chain Monte Carlo (MCMC) procedure (see Materials and Methods). An important insight is that, in this case, the conformational sampling method uniquely defines the reference state. Thus, in the case of a fragment library, the reference distribution 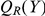 is the probability distribution over 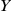 that is obtained by sampling conformations solely using the fragment library.

As the method we have introduced here invariably relies on the ratio of two probability distributions – one regarding protein structure and the other regarding a well-defined reference state – we refer to it as the *reference ratio method*. In the next section, we show that the standard pairwise distance PMFs can be seen as an approximation of the reference ratio method.

### Pairwise distance PMFs explained

In this section, we apply the reference ratio method to the standard, pairwise distance case. In the classic PMF approach, one considers the vector of pairwise distances 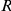 between the amino acids. In this case, it is usually assumed that we can write
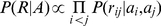
(7)where the product runs over all amino acid pairs 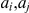 (with 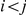), and 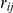 is their matching distance. Clearly, the assumption that the joint probability can be written as a product of pairwise probabilities is not justified [12], [13], [43], but in practice this assumption often provides useful results [22]. In order to obtain protein-like conformations, 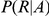 needs to be combined with an appropriate probability distribution 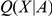 that addresses the local features of the polypeptide chain. Applying Eq. 6 to this case results in the following expression:
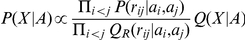
where the denominator 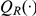 is the probability distribution over the pairwise distances as induced by the distribution 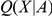. The ratio in this expression corresponds to the probabilistic expression of a PMF. The reference state is thus determined by 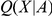: it reflects the probability of generating a set of pairwise distances using local structure information alone. Obviously, as 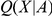 is conditional upon the amino acid sequence 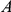, the reference state becomes sequence dependent as well.

We again emphasize that the assumption of pairwise decomposability in Eq. 7 is incorrect [12]–[14], [43]. Therefore, the application of the reference ratio method results in a useful approximation, at best. As a result, the optimal definition of the reference state also needs to compensate for the errors implied by the invalid assumption. As is it well established that distance dependent PMFs perform well with a suitable definition of the reference state [3], [22], [38]–[40], and the incorrect pairwise decomposability assumption impairs a rigorous statistical analysis, we do not discuss this type of PMFs further. Indeed, for pairwise distance PMFs, the main challenge lies in developing better probabilistic models of sets of pairwise distances [49].

The pairwise distance PMFs currently used in protein structure prediction are thus not statistically rigorous, because they do not make use of a proper joint probability distribution over the pairwise distances, which are strongly intercorrelated due to the connectivity of molecules. A rigorous application of the reference ratio method would require the construction of a proper joint probability distribution over pairwise distances. This is certainly possible in principle, but currently, as far as we know, a challenging open problem and beyond the scope of this article. However, we have clarified that the idea of using a reference state is correct and valid, and that this state has a very precise definition. Therefore, in the next two sections, we show instead how statistically valid quantities, similar to PMFs, can be obtained for very different coarse grained variables.

### A generalized PMF: radius of gyration

As a first application of the reference ratio method, we consider the task of sampling protein conformations with a given probability distribution 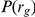 for the radius of gyration 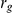. For 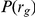, we chose a Gaussian distribution with mean 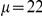 Å and standard deviation 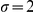 Å. This choice is completely arbitrary; it simply serves to illustrate that the reference ratio method allows imposing an exact probability distribution over a certain feature of interest. Applying Eq. 6 results in:
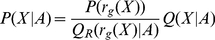
(8)For 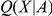, we used TorusDBN – a graphical model that allows sampling of plausible backbone angles [45] – and sampled conditional on the amino acid sequence 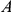 of ubiquitin (see Materials and Methods). 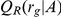 is the probability distribution of the radius of gyration for structures sampled solely from TorusDBN, which was determined using generalized multihistogram MCMC sampling (see Materials and Methods).

**Figure 3:**
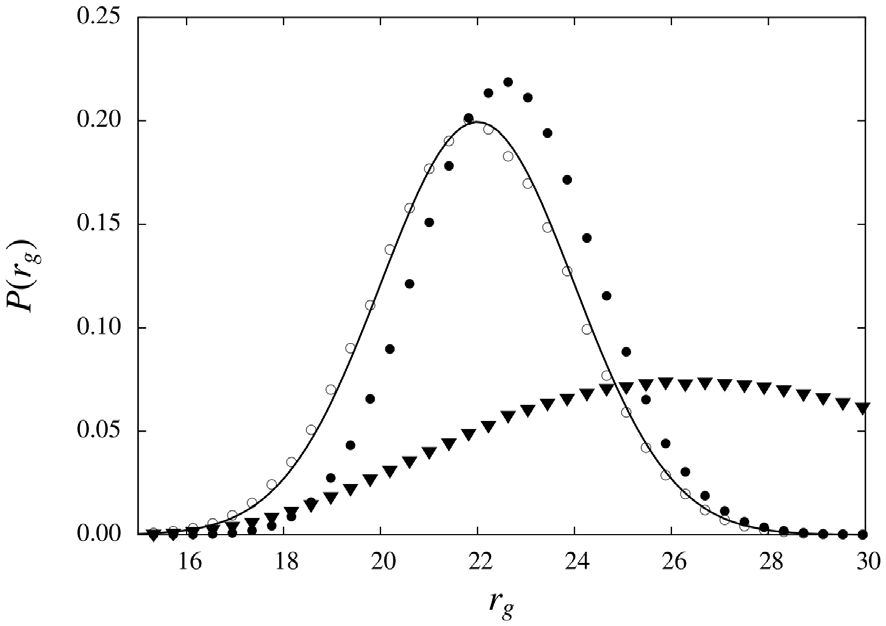
A PMF based on the radius of gyration. The goal is to adapt a distribution 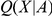 – which allows sampling of local structures – such that a given target distribution 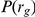 is obtained. For 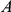, we used the amino acid sequence of ubiquitin. Sampling from 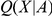 alone results in a distribution with an average 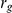 of about 27 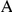 (triangles). Sampling using the correct expression (open circles), given by Eq. 8, results in a distribution that coincides with the target distribution (solid line). Not taking the reference state into account results in a significant shift towards higher 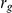 (black circles).

In Fig. 3, we contrast sampling from Eq. 8 with sampling from 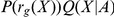. In the latter case, the reference state is not properly taken into account, which results in a significant shift towards higher radii of gyration. In contrast, the distribution of 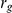 for the correct distribution 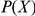, given by Eq. 8, is indistinguishable from the target distribution. This qualitative result is confirmed by the Kullback-Leibler divergence [50] – a natural distance measure for probability distributions expressed in bits – between the target distribution and the resulting marginal distributions of 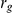. Adding 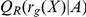 to the denominator diminishes the distance from 0.08 to 0.001 bits. For this particular PMF, the effect of using the correct reference state is significant, but relatively modest; in the next section, we discuss an application where its effect is much more pronounced.

### Iterative optimization of PMFs: hydrogen bonding

Here, we demonstrate that PMFs can be optimized iteratively, which is particularly useful if the reference probability distribution 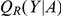 is difficult to estimate. We illustrate the method with a target distribution that models the hydrogen bonding network using a multinomial distribution.

We describe the hydrogen bonding network (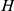) with eight integers (for details, see Materials and Methods). Three integers 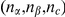 represent the number of residues that do not partake in hydrogen bonds in 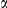-helices, 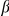-sheets and coils, respectively. The five remaining integers 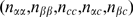 represent the number of hydrogen bonds within 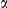-helices, within 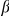-strands, within coils, between 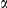-helices and coils, and between 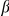-strands and coils, respectively.

As target distribution 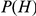 over these eight integers, we chose a multinomial distribution whose parameters were derived from the native structure of protein G (see Materials and Methods). 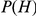 provides information, regarding protein G, on the number of hydrogen bonds and the secondary structure elements involved, but does not specify *where* the hydrogen bonds or secondary elements occur. As in the previous section, we use TorusDBN as the sampling distribution 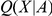; we sample backbone angles conditional on the amino acid sequence 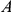 of protein G. Native secondary structure information was *not* used in sampling from TorusDBN.

The reference distribution 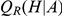, due to TorusDBN, is very difficult to estimate correctly for several reasons: its shape is unknown and presumably complex; its dimensionality is high; and the data is very sparse with respect to 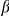-sheet content. Therefore, 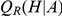 can only be approximated, which results in a suboptimal PMF. A key insight is that one can apply the method iteratively until a satisfactory PMF is obtained (see Fig. 2, dashed line). In each iteration, the (complex) reference distribution is approximated using a simple probability distribution; we illustrate the method by using a multinomial distribution, whose parameters are estimated by maximum likelihood estimation in each iteration, using the conformations generated in the previous iteration. In the first iteration, we simply set the reference distribution equal to the uniform distribution.

Formally, the procedure works as follows. In iteration 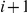, the distribution 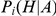 is improved using the samples generated in iteration 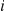:
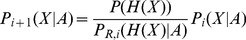
(9)where 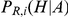 is the reference distribution estimated from the samples generated in the 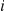-th iteration, 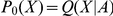 stems from TorusDBN, and 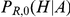 is the uniform distribution. After each iteration, the set of samples is enriched in hydrogen bonds, and the reference distribution 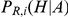 can be progressively estimated more precisely. Note that in the first iteration, we simply use the product of the target and the sampling distribution; no reference state is involved.

**Figure 4:**
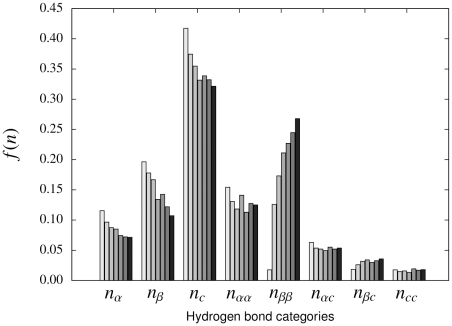
Iterative estimation of a PMF. For each of the eight hydrogen bond categories (see text), the black bar to the right denotes the fraction of occurrence 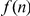 in the native structure of protein G. The gray bars denote the fractions of the eight categories in samples from each iteration; the first iteration is shown to the left in light gray. In the last iteration (iteration 6; dark gray bars, right) the values are very close to the native values for all eight categories. Note that hydrogen bonds between 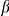-strands are nearly absent in the first iteration (category 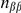).

**Figure 5:**
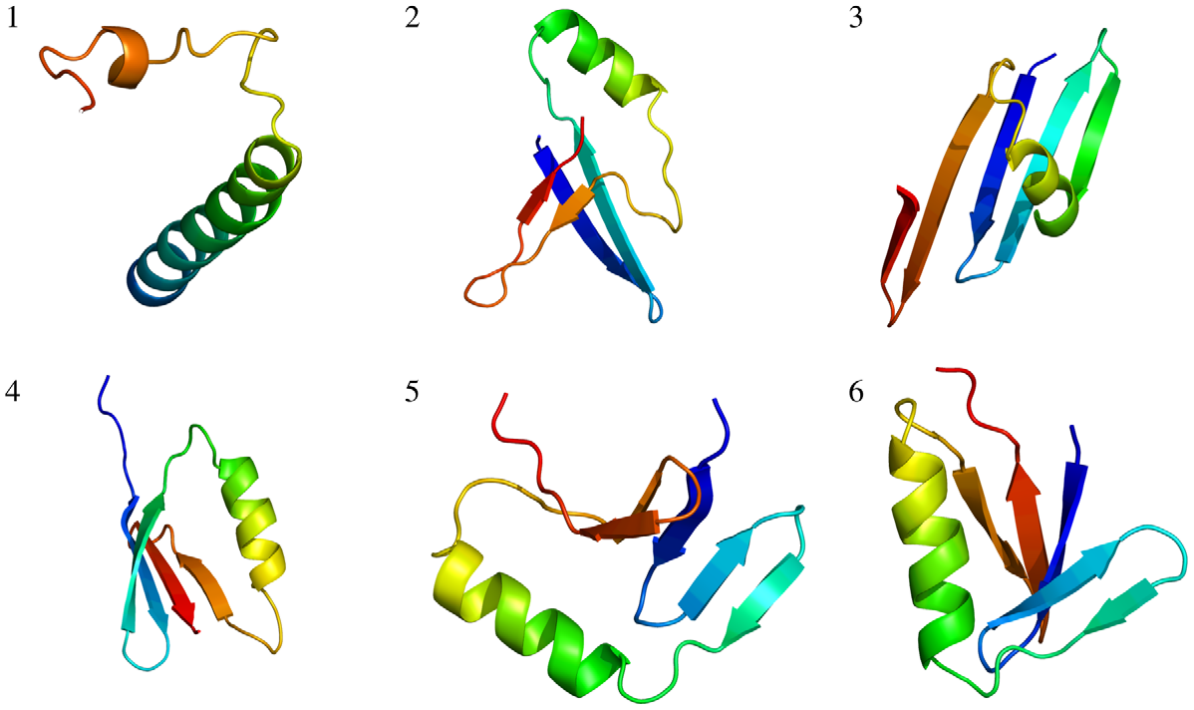
Highest probability structures for each iteration. The structures with highest probability out of 50,000 samples for all six iterations (indicated by a number) are shown as cartoon representations. The N-terminus is shown in blue. The figure was made using PyMOL [64].

Fig. 4 shows the evolution of the fractions versus the iteration number for the eight hydrogen bond categories; the structures with minimum energy for all six iterations are shown in Fig. 5. In the first iteration, the structure with minimum energy (highest probability) consists of a single 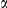-helix; 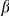-sheets are entirely absent (see Fig. 5, structure 1). Already in the second iteration, 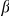-strands start to pair, and in the third and higher iterations complete sheets are readily formed. The iterative optimization of the PMF quickly leads to a dramatic enrichment in 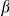-sheet structures, as desired, and the fractions of the eight categories become very close to the native values (Fig. 4).

### Conclusions

The strengths and weaknesses of PMFs can be rigorously explained based on simple probabilistic considerations, which leads to some surprising new insights of direct practical relevance. First, we have made clear that PMFs naturally arise when two probability distributions need to be combined in a meaningful way. One of these distributions typically addresses local structure, and its contribution often arises from conformational sampling. Each conformational sampling method thus requires its own reference state and corresponding reference distribution; this is likely the main reason behind the large number of different reference states reported in the literature [3], [22], [38]–[41]. If the sampling method is conditional upon the amino acid sequence, the reference state necessarily also depends on the amino acid sequence.

Second, conventional applications of pairwise distance PMFs usually lack two necessary features to make them fully rigorous: the use of a proper probability distribution over pairwise distances in proteins for 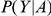, and the recognition that the reference state is rigorously defined by the conformational sampling scheme used, that is, 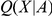. Usually, the reference state is derived from external physical considerations [11], [51].

Third, PMFs are not tied to pairwise distances, but generalize to any coarse grained variable. Attempts to develop similar quantities that, for example, consider solvent exposure [52], [53], relative side chain orientations [54], backbone dihedral angles [55], [56] or hydrogen bonds [37] are thus, in principle, entirely justified. Hence, our probabilistic interpretation opens up a wide range of possibilities for advanced, well-justified energy functions based on sound probabilistic reasoning; the main challenge is to develop proper probabilistic models of the features of interest and the estimation of their parameters [49], [57]. Strikingly, the example applications involving radius of gyration and hydrogen bonding that we presented in this article *are* statistically valid and rigorous, in contrast to the traditional pairwise distance PMFs.

Finally, our results reveal a straightforward way to optimize PMFs. Often, it is difficult to estimate the probability distribution that describes the reference state. In that case, one can start with an approximate PMF, and apply the method iteratively. In each iteration, a new reference state is estimated, with a matching probability distribution. In that way, one iteratively attempts to sculpt an energy funnel [24], [32]–[36]. We illustrated this approach with a probabilistic model of the hydrogen bond network. Although iterative application of the inverse Boltzmann formula has been described before [24], [35], [58], [59], its theoretical justification, optimal definition of the reference state and scope remained unclear.

As the traditional pairwise distance PMFs used in protein structure prediction arise from the imperfect application of a statistically valid and rigorous procedure with a much wider scope, we consider it highly desirable that the name “potential of mean force” should be reserved for true, physically valid quantities [13]. Because the statistical quantities we discussed invariably rely on the use of a ratio of two probability distributions, one concerning protein structure and the other concerning the (now well defined) reference state, we suggest the name “reference ratio distribution” deriving from the application of the “reference ratio method”.

Pairwise distance PMFs, as used in protein structure prediction, are not physically justified potentials of mean force or free energies [2], [13] and the reference state does not depend on external physical considerations; the same is of course true for our generalization. However, these PMFs are approximations of statistically valid and rigorous quantities, and these quantities can be generalized beyond pairwise distances to other aspects of protein structure. The fact that these quantities are not potentials of mean force or free energies is of no consequence for their statistical rigor or practical importance – both of which are considerable. Our results thus vindicate, formalize and generalize Sippl's original and seminal idea [7]. After about twenty years of controversy, PMFs – or rather the statistical quantities that we have introduced in this article – are ready for new challenges.

## Materials and Methods

### Outline of the problem

We consider a joint probability distribution 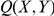 and a probability distribution 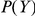 over two variables of interest, 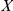 and 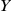, where 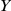 is a deterministic function of 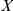; we write 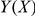 when relevant. Note that because 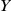 is a function of 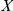, it follows that 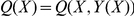; and if 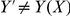, then 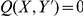.

We assume that 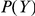 is a meaningful and informative distribution for 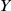. Next, we note that 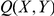 implies a matching marginal probability distribution 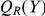 (where the subscript 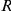 refers to the fact that 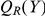 corresponds to the reference state, as we will show below):
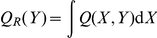
We consider the case where 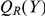 differs substantially from 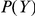; hence, 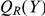 can be considered as incorrect. On the other hand, we also assume that the conditional distribution 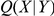 is indeed meaningful and informative (see next section). This distribution is given by:
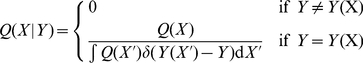
(10)where 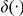 is the delta function. The question is now how to combine the two distributions 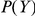 and 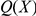 – each of which provide useful information on 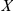 and 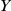 – in a meaningful way. Before we provide the solution, we illustrate how this problem naturally arises in protein structure prediction.

### Application to protein structure

In protein structure prediction, 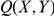 is often embodied in a fragment library; in that case, 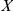 is a set of atomic coordinates obtained from assembling a set of polypeptide fragments. Of course, 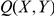 could also arise from a probabilistic model, a pool of known protein structures, or any other conformational sampling method. The variable 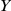 could, for example, be the radius of gyration, the hydrogen bond network or the set of pairwise distances. If 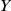 is a deterministic function of 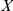, the two variables are called *coarse grained* and *fine grained* variables, respectively. For example, sampling a set of dihedral angles for the protein backbone uniquely defines the hydrogen bond geometry between any of the backbone atoms.

Above, we assumed that 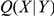 is a meaningful distribution. This is often a reasonable assumption; fragment libraries, for example, originate from real protein structures, and conditioning on protein-like compactness or hydrogen bonding will thus result in a meaningful distribution. Of course, sampling solely from 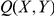 is not an efficient strategy to obtain hydrogen bonded or compact conformations, as they will be exceedingly rare. We now provide the solution of the problem outlined in the previous section, and discuss its relevance to the construction of PMFs.

### Solution for a proper joint distribution

A first step on the way to the solution is to note that the product rule of probability theory allows us to write:
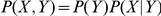
As only 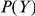 is given, we need to make a reasonable choice for 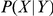. We assume, as discussed before, that 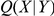 is a meaningful choice, which leads to:
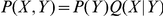
In the next step, we apply the product formula of probability theory to the second factor 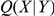, and obtain:
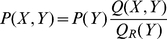
(11)The distribution 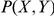 has the correct marginal distribution 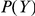.

In the next two sections, we discuss how this straightforward result can be used to great advantage for understanding and generalizing PMFs. First, we show that the joint distribution specified by Eq. 11 can be reduced to a surprisingly simple functional form. Second, we discuss how this result can be used in MCMC sampling. In both cases, expressions that correspond to a PMF arise naturally.

### PMFs from combining distributions

Using the product rule of probability theory, Eq. 11 can be written as:
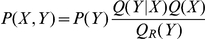
Because the coarse grained variable 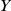 is a deterministic function of the fine grained variable 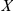, 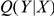 is the delta function:
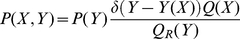
(12)Finally, we integrate out the, now redundant, coarse grained variable 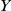 from the expression:
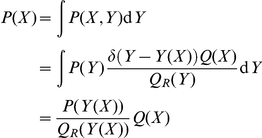
and obtain our central result (Eq. 6). Sampling from 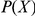 will result in the desired marginal probability distribution 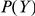. The influence of the fine grained distribution 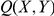 is apparent in the fact that 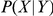 is equal to 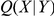. The ratio in this expression corresponds to the usual probabilistic formulation of a PMF; the distribution 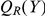 corresponds to the reference state. In the next section, we show that PMFs also naturally arise when 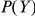 and 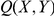 are used together in Metropolis-Hastings sampling.

### PMFs from Metropolis-Hastings sampling

Here, we show that Metropolis-Hastings sampling from the distribution specified by Eq. 11, using 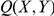 as a proposal distribution, naturally results in expressions that are equivalent to PMFs. The derivation is also valid if the proposal distribution depends on the previous state, provided 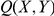 satisfies the detailed balance condition.

According to the standard Metropolis-Hastings method [60], one can sample from a probability distribution 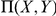 by generating a Markov chain where each state 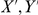 depends only on the previous state 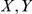. The new state 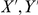 is generated using a proposal distribution 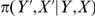, which includes 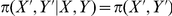 as a special case. According to the Metropolis-Hastings method, the proposal 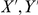 is accepted with a probability 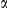:
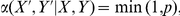


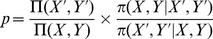
(13)where 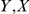 is the starting state, and 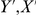 is the next proposed state. We assume that the proposal distribution 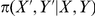 satisfies the detailed balance condition:
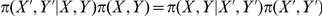
As a result, we can always write Eq. 13 as:
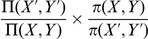
The Metropolis-Hastings expression (Eq. 13), applied to the distribution specified by Eq. 11 and using 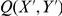 or 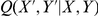 as the proposal distribution, results in:
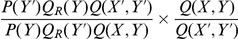
which reduces to:
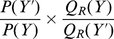
(14)Hence, we see that the Metropolis-Hastings method requires the evaluation of ratios of the form 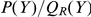 when 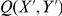 or 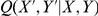 is used as the proposal distribution; these ratios correspond to the usual probabilistic formulation of a PMF. Finally, when 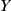 is a deterministic function of 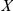, the proposal distribution reduces to 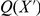 or 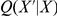, and Eq. 14 becomes:
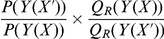



### Application to radius of gyration and hydrogen bonding

Conformational sampling from a suitable 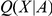 was done using TorusDBN [45] as implemented in Phaistos [61]; backbone angles (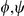 and 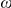) were sampled conditional on the amino acid sequence. We used standard fixed bond lengths and bond angles in constructing the backbone coordinates from the angles, and represented all side chains (except glycine and alanine) with one dummy atom with a fixed position [61].

For the radius of gyration application, we first determined 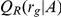 using the multi-canonical MCMC method to find the sampling weights 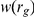 that yield a flat histogram [62]. Sampling from the resulting joint distribution (Eq. 8) was done using the same method. In both cases, we used 50 million iterations; the 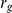 bin size was 0.08 Å. Sampling from TorusDBN was done conditional on the amino acid sequence 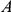 of ubiquitin (76 residues, PDB code 1UBQ).

For the hydrogen bond application, sampling from the PMFs was done in the 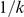-ensemble [63], using the Metropolis-Hastings algorithm and the generalized multihistogram method for updating the weights [62]. In each iteration 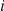, 50,000 samples (out of 50 million Metropolis-Hastings steps) were generated, and the parameters of the multinomial distribution 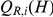 were subsequently obtained using maximum likelihood estimation. Hydrogen bonds were defined as follows: the 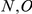 distance is below 3.5 Å, and the angles formed by 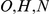 and 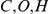 are both greater than 100. Each carbonyl group was assumed to be involved in at most one hydrogen bond; in case of multiple hydrogen bond partners, the one with the lowest 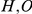 distance was selected. Each residue was assigned to one of the eight possible hydrogen bond categories 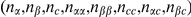 based on the presence of hydrogen bonding at its carbonyl group and the secondary structure assignments (for both bond partners) by TorusDBN. The target distribution – the multinomial distribution 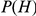 used in Eq. 9 – was obtained by maximum likelihood estimation using the number of hydrogen bonds, for all eight categories, in the native structure of protein G (56 residues, PDB code 2GB1). Sampling from TorusDBN was done conditional on the amino acid sequence of protein G; native secondary structure information was *not* used.
